# Paediatric CT made easy

**DOI:** 10.1007/s00247-022-05526-0

**Published:** 2022-11-05

**Authors:** Eszter Nagy, Sebastian Tschauner, Clemens Schramek, Erich Sorantin

**Affiliations:** Division of Paediatric Radiology, Department of Radiology, Medical University of Graz, Auenbruggerplatz 34, 8036 Graz, Austria

**Keywords:** ALARA, Children, Computed tomography, Image Gently, Imaging, Radiation protection

## Abstract

Paediatric computed tomography (CT) imaging has always been associated with challenges. Although the technical background of CT imaging is complex, it is worth considering the baseline aspects of radiation exposure to prevent unwanted excess radiation in paediatric patients. In this review, we discuss the most relevant factors influencing radiation exposure, and provide a simplified and practical approach to optimise paediatric CT.

## Introduction

Anatomical and physiological differences between children and adults are more pronounced the younger a patient is. Several of these differences can be explained by the necessity of a higher metabolic rate in children to ensure growth (e.g., double oxygen demand per kilogram body weight), which leads to higher respiratory and heart rates. Bones have higher cartilaginous content and there is less retroperitoneal fat, which results in less inherent tissue contrast. Growth means higher cell proliferation with more cells in mitosis, exhibiting higher radiation sensitivity. This is aggravated by the fact that in infants, 25% of red bone marrow is in skull bones, thus making the relative radiation sensitivity of the head three times higher compared to adults [1]. The relatively large intravascular volume (90 ml/kg body weight in infants vs. 70 ml/kg body weight in adults) and the faster circulation due to a higher heart rate are well-known challenges when injecting intravenous contrast media. These factors are transforming the intravenous contrast media administration to a “hit or miss” event. Therefore, computed tomography (CT) scanners with high temporal and soft-tissue resolution are required and a careful adjustment of all relevant factors affecting radiation exposure is recommended.

In this article, we review the most relevant factors influencing radiation exposures and the resulting image quality and present a practical approach to paediatric CT imaging.

## Relevant technical parameters and their relationship with radiation exposure and image quality

**Fig. 1:**
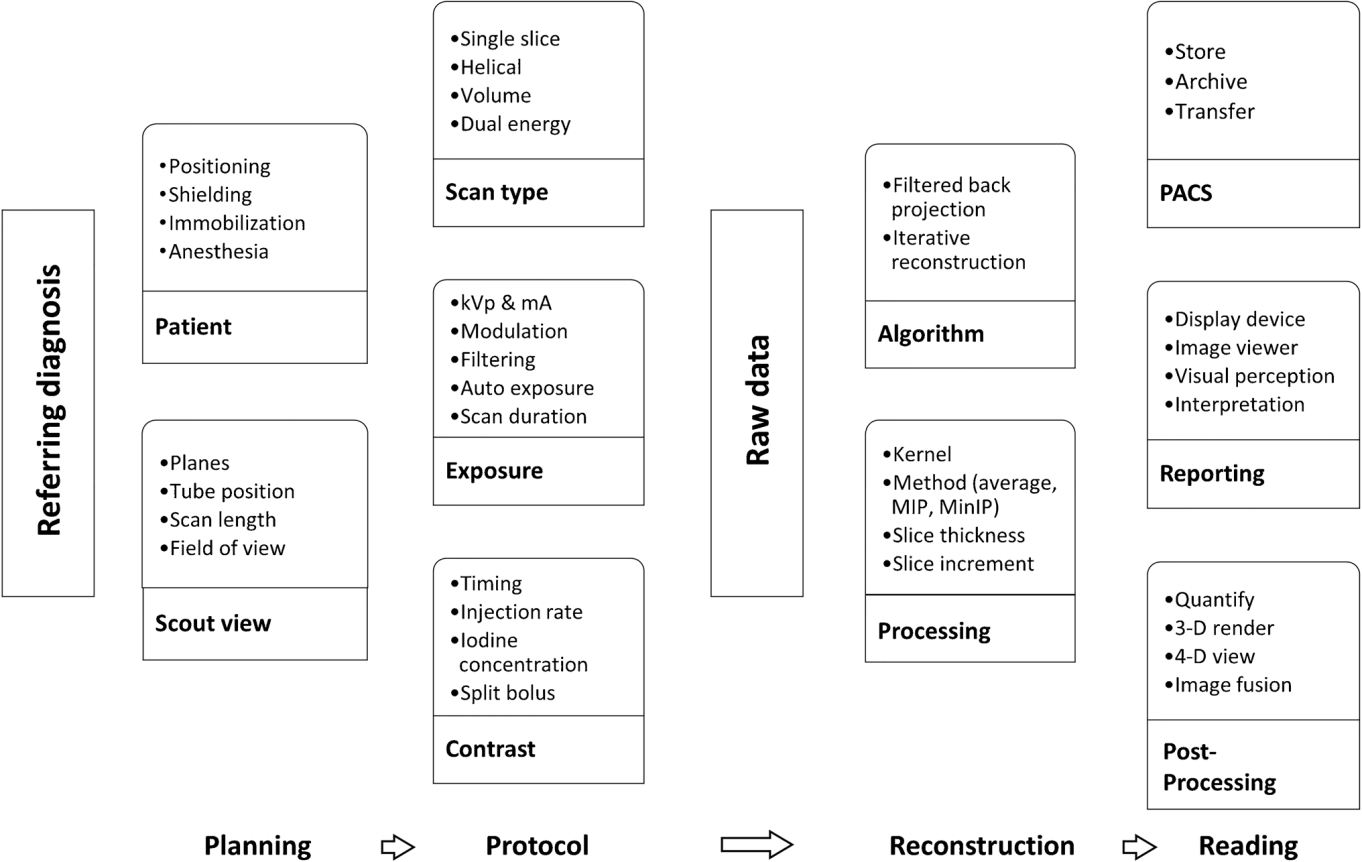
The graphic depicts the imaging chain. This review focuses on the first three columns (planning the examination, image acquisition and image reconstruction)

The main goal when optimising CT protocols is to achieve diagnostic image quality at the lowest possible radiation exposure. It is important to note that diagnostic image quality is not equivalent to perfect image quality and depends on the clinical question. The most important descriptor of image quality is image noise. In the following section, we introduce a practical approach to paediatric CT imaging, highlighting the most relevant factors when considering radiation exposure and the resulting image quality (Fig. 1).

### Clinical referral

Every examination starts with a clinical referral, which should include the clinical question and the body region to be scanned. It needs to be emphasised that the clinical referral represents the central factor responsible for the scan protocol and therefore influences the final radiation exposure. Depending on the clinical question, scans may be performed with reduced exposure settings. As a practical tool, image quality scoring criteria were introduced to evaluate the image quality based on clinical indications [2]. From the point of view of radiation protection and noninvasive imaging, the best radiation protection is NOT to perform a CT examination. According to the ALARA (as low as reasonably achievable) principle and the derived Image Gently [3] and Eurosafe Imaging Campaign [4], paediatric radiologists must participate in the management of children in order to select the best-suited imaging modality, which answers the query of referring physicians at the lowest achievable radiation exposure. Standards of the European Union and international guidelines may also guide clinical decision-making [5, 6]. Note that clinical decisions should always be made on an individual basis. In many cases, a CT examination is avoidable with alternative imaging possibilities being appropriate. For this reason, discussion between paediatricians and radiologists should be encouraged, particularly when it is not clear which imaging modality should be chosen. To facilitate this, the “Paediatric Radiologist of the Day” was introduced at the authors’ institution. One paediatric radiologist is on call to discuss any questions with the consultant paediatrician in charge. This approach shortens communication and saves time for all colleagues.

Practically, body weight, height and the results of kidney function tests should be noted, the latter is mandatory if intravenous contrast medium is necessary. Moreover, information about implants (e.g., pacemakers, since not all have a licence for CT scanning) and comorbidities (e.g., thyroid disease) should either be included with the referral or discussed with the paediatrician.

### Patient positioning

Operators are responsible for the correct positioning of the patient in the isocentre of the gantry. Off-centre scanning results in misalignment with respect to the bowtie filter, which negatively affects the radiation distribution. If the patient is moved closer to the x-ray tube, their silhouette will appear larger and therefore radiation exposure will be higher due to automated exposure control (AEC). On the other hand, if the patient lies farther from the tube, their silhouette will appear smaller and therefore AEC will regulate radiation exposure down, thus degrading image quality by increasing noise. Off-centred patient positioning can result in up to 50% exposure differences as compared to correct central positioning [7]. Artificial intelligence-based systems are now available to ensure correct central positioning [8].

**Fig. 2:**
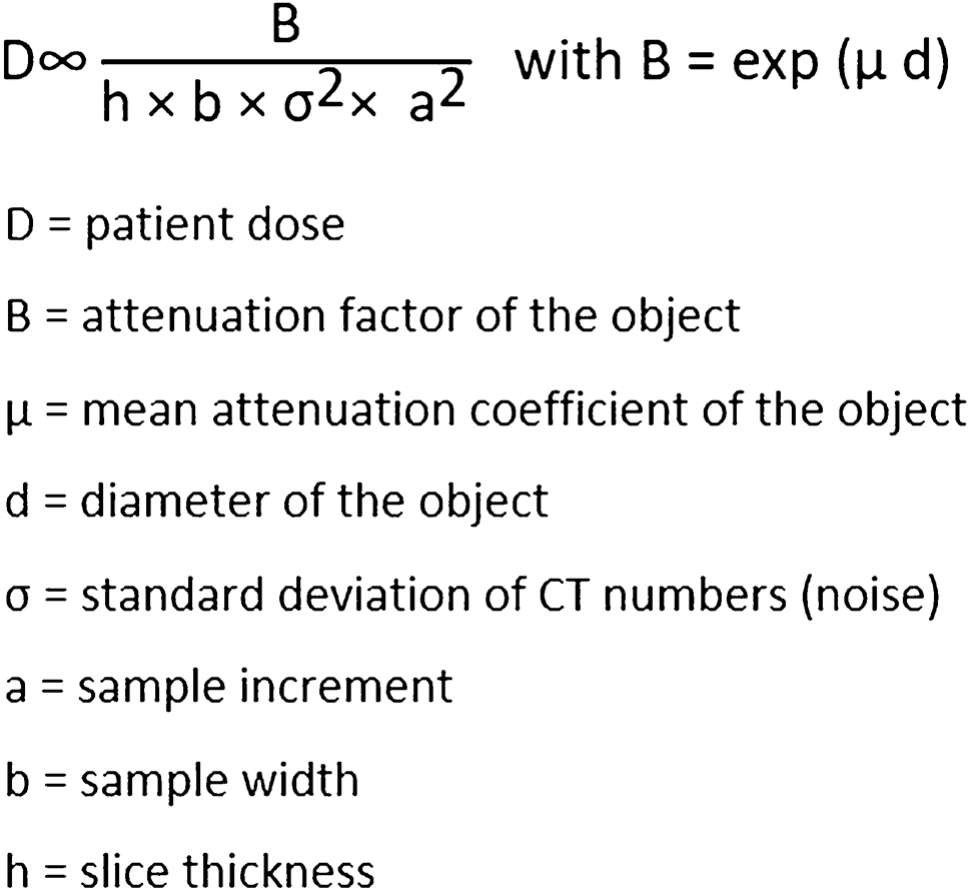
Brooks formula, where D stands for patient dose, B for attenuation factor of the object, µ for mean attenuation coefficient of the object, d for diameter of the object, σ for standard deviation of Hounsfield units within a region of interest (image noise), a for sample increment, b for sample width and h for slice thickness

In addition to centring, the position of the arms also influences radiation exposure. Ideally for chest examinations, the patient should be examined with their hands above their head. If the patient is unwilling or unable (e.g., a fracture of the upper extremity) to do this, the best solution is to cross their hands in front of their body with stretched arms and place some towels between their arms and trunk. With this approach, the anteroposterior (AP) diameter is increased and patient geometry is more rounded, which results in a more homogenous radiation distribution and less beam-hardening artefact. The physics of this is explained by Brooks formula, which describes the link between radiation exposure and image noise (Fig. 2) [9]. According to this formula, if patient diameter increases by 4 cm (e.g., their hand is on the side of the body), the radiation exposure must be doubled to maintain image quality.

**Fig. 3:**
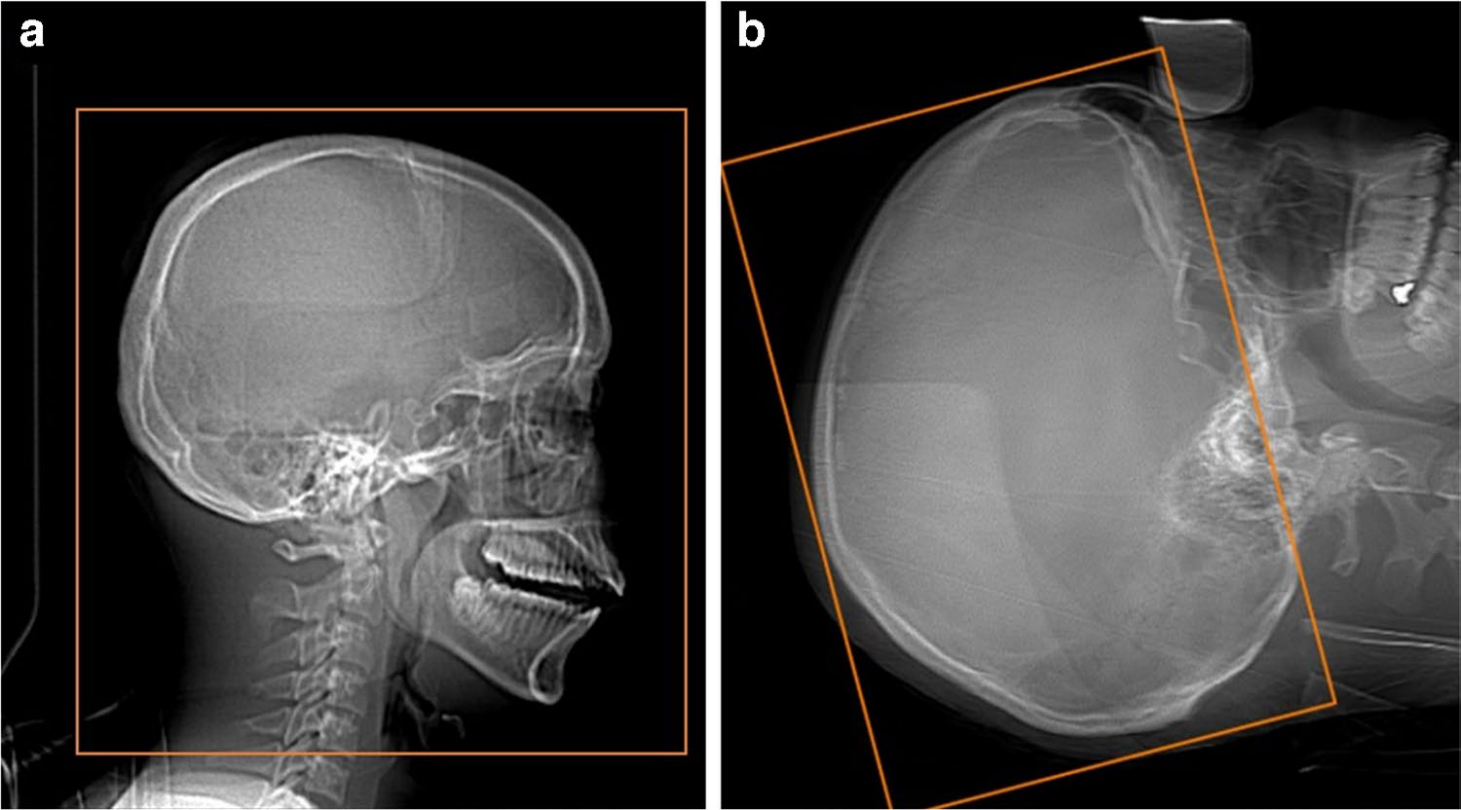
A computed tomography scan in the trauma setting without (**a**) and with (**b**) gantry tilting

There are also considerations for head positioning, which may impact radiation exposure. Ideally, the head is in a head-holder ensuring the most optimal radiation exposure. If a head-holder is not possible, table configuration and the mass below the head should be considered, which might affect AEC and increase radiation exposure. To reduce or avoid ocular lens exposure in a head scan, the scanning angle should be parallel to a line created by the supraorbital ridge and the inner table of the posterior margin of the foramen magnum. This may be achieved by tilting the gantry (Fig. 3). However, if the cervical spine is also to be examined, the scan should be performed with an extended neck, without gantry tilting. Otherwise, the chin would be located above the thyroid, resulting in an increased radiation exposure of the latter.

### CT localiser radiographs

The next step in the imaging chain is the localiser radiograph (scout view, topogram, etc.). Usually, two scout images are performed (AP or posteroanterior [PA] and lateral) to ensure the correct patient positioning in the gantry isocentre. The PA view is preferred over the AP view, since this means significant reduction of radiation exposure to the radiosensitive organs such as the breasts and thyroid [10]. However, the overall radiation exposure of the scout view might increase with the PA view since high-density structures (ribs, vertebra) will be closer to the tube [11]. Notably, in younger patients, there is less red bone marrow within these areas, thus decreasing regional radiosensitivity [12]. Meticulous adaption of exposure parameters to patient size is required to outweigh this possible increment in radiation exposure. The length of the scout and scan should be adjusted to the clinical question to avoid overscanning. For low-dose CT scans, scout images increasingly account for a significant proportion (up to one-fifth) of the radiation dose of the study [13].

### Additional tube filtering

X-ray tubes emit a spectrum of different tube voltages, up to the peak kilovoltage (kVp). In most cases, lower-energy spectra are not considered useful for imaging. Indeed, low kilovoltages are typically scattered and absorbed in the body without ever reaching the detector. In high-contrast scenarios (air to soft tissue = lung, or soft tissue to bone = musculoskeletal), additional tin filtering has proven to effectively reduce radiation exposure. However, this feature is available on only a limited number of scanners and only at certain kV values (e.g., more than 100 kV).

### Shielding

Patient shielding is a related topic to tube filtering and has been actively discussed with many uncertainties and controversies. To overcome these issues, a European recommendation was recently published [14]. According to this consensus document, in most cases there is no need for in-plane or out-of-plane shielding when using up-to-date CT technology. The current radiation exposure reduction with organ dose modulation may outperform the radiation reduction effects of shielding without the increased risk of disturbing image artefacts, infection and patient discomfort. Nevertheless, if in-plane shielding is applied, the operator should place the shields after the scout views to avoid interference with AEC.

### Scanning type

Paediatric CT is performed mainly in spiral (or helical) or volume (or axial) scan mode. While spiral CT is burdened by the over-ranging and over-beaming effect, over-beaming does not play a role in volume CT, except for multiple overlapping rotations.

Over-ranging is defined as an extension of scan range by half a gantry rotation, which is required for reconstruction of the boundaries of the image. Over-ranging is directly proportional to collimation width and pitch. Since over-ranging is independent of scan length, the relative radiation exposure arising from over-ranging is higher if the length of the scan is short (e.g., paediatric chest) [15]. Some manufacturer-specific solutions such as dynamic beam collimation or hybrid reconstruction algorithms have been developed to reduce the radiation exposure due to over-ranging [16].

Over-beaming is a redundant radiation exposure per rotation and inversely proportional to the number of detector rows. Therefore, multi-detector CT with wide detectors should be applied in children, since the over-beaming effect is not significant with these scanners.

Due to detector configuration in recent CT scanners, single-slice mode is no longer available. This is because small volumes must be scanned in every case. Therefore, this scan mode can be considered outdated.

Modern single-source and dual-source CT (DSCT) scanners also offer dual-energy scanning mode. Experience with paediatric patients is, however, still limited, but studies suggest that DSCT exposes children to comparable or even lower radiation exposure than single-energy CT [17]. In our experience, single-source dual-energy scanning requires about 30% higher radiation exposure than the single-energy mode, which makes this scanning mode rather unattractive for small patients. The only indication for dual-energy single-source scans in our institution is for metal-artefact reduction in extremity CTs [18].

### Exposure settings

The relationship between tube current (in mAs) and radiation exposure is linear, which means doubling mAs results in double radiation exposure (assuming all other factors are constant). Increasing mAs means less image noise and therefore better image quality, while image contrast is not affected.

**Fig. 4:**
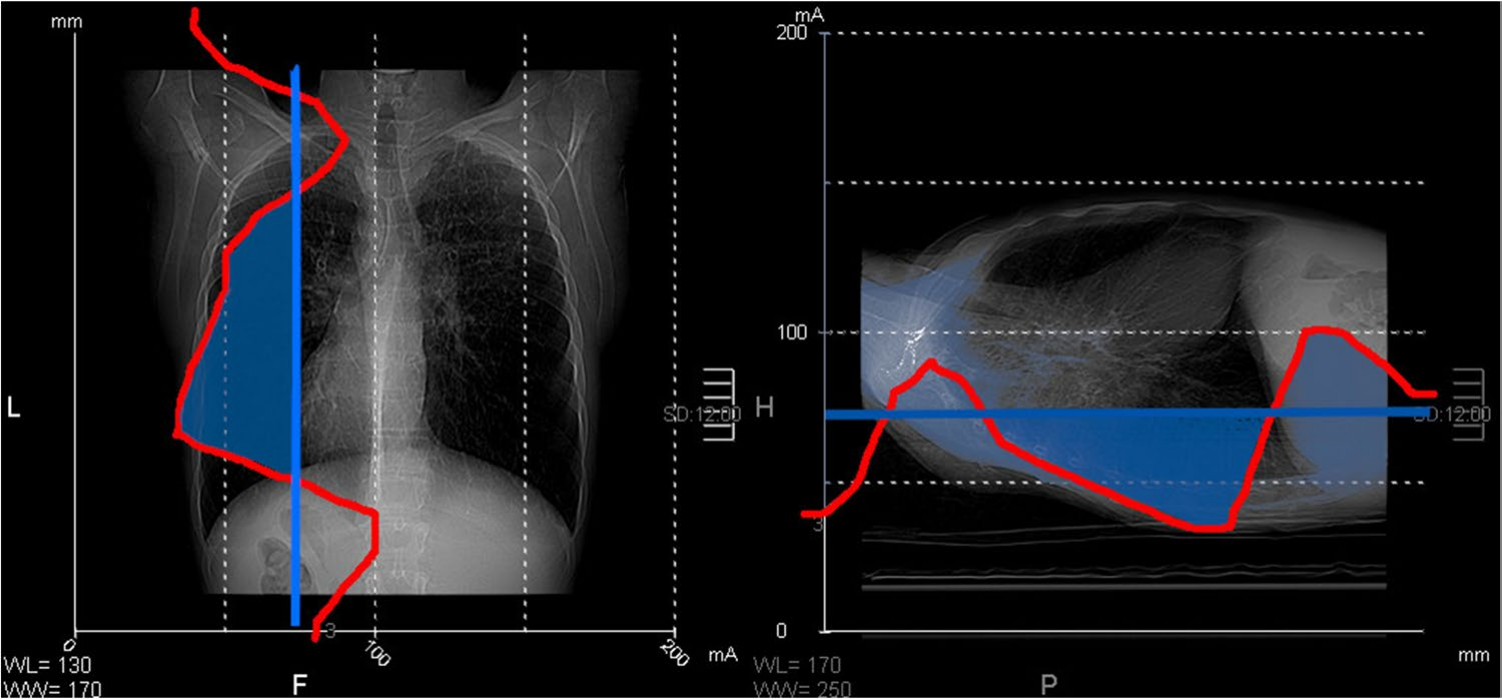
Modulation of radiation exposure with automated exposure control (AEC). The red line represents the attenuation profile of the body. The light blue line represents the selected minimum value for AEC. If the blue line is not set sufficiently low, as in this case, then there is an excess radiation exposure in thoracic region, indicated by the blue area (adapted from [10])

AEC systems are commonly advised for radiation exposure reduction [19]. AEC systems assess the x-ray attenuation profile of the body in the scan range based on the scout view and adjust the tube current to maintain image quality during the entire scan. Different AEC systems define image quality in various ways; “image noise” and “noise index” are used by Canon (formerly Toshiba) and GE Healthcare, respectively, while “reference mAs” is used by Siemens [11]. The operator selects the minimum and maximum levels of mAs for the AEC. It is advisable to set the minimum level to the lowest value to achieve the best possible reduction in radiation exposure (Fig. 4).

Tube voltage (kV) also strongly determines radiation dose. The relationship between kV and radiation exposure is quadratic, which means a more than 50% reduction in radiation exposure if tube voltage is reduced from 120 kV to 80 kV and all other dose-relevant parameters are kept constant. On the other hand, the reduced kV will result in greater attenuation differences in tissues, based on the photoelectric effect, and thus result in higher intrinsic contrast [20]. This phenomenon also reduces the volume of contrast agent administered [21]. However, imaging with reduced tube voltage only benefits small patients. With increasing patient size, the images are more burdened by the beam-hardening effect, which deteriorates diagnostic image quality. Unfortunately, there is no clear cutoff regarding patient size and the extent of kV reduction, and it may vary from scanner to scanner. In our practice, tube voltage is defined by effective patient diameter (the square root of the sum of AP and lateral patient diameter), which can be calculated from the scout views.

As mentioned previously, lower kV leads to increased inherent tissue contrast and to a shift to higher Hounsfield unit values. A discrepancy between tube voltages of the bolus tracking monitoring scan and the following angiography may result in an inappropriate scan start based on the altered tissue contrast. Therefore, tube voltage for the bolus tracking and for the diagnostic angiography must be the same and the bolus tracking threshold must be adjusted to the altered tube voltage [22].

Based on Brooks formula, slice thickness is inversely related to radiation exposure. Therefore, half-slice thickness needs double radiation exposure for the same image noise, which mainly defines image quality [10]. To avoid a partial volume effect, the increment should be 50%.

Pitch is defined as table travel per rotation divided by the beam collimation. A higher pitch results in a faster scan and consequently less motion artefact, which might avoid general anaesthesia for DSCT [22, 23]. Notably, in case of increased pitch, the scanner might increase tube current automatically to avoid impaired image quality and the over-ranging effect may increase [24]. Therefore, it is recommended to avoid a pitch higher than 1.5 in single-source CT and pitch should be maximized at − 3.4 in DSCT. Modern CT scanners select optimal pitch automatically as determined by all other scanning parameters, thus minimising operator input.

### Contrast medium

Contrast agent administration in CT dictates additional consideration for patient radiation burden. Bolus tracking is commonly recommended in paediatric patients for angiography. For chest CT angiography, the bolus tracking monitoring scan is usually in the range of the radiosensitive breasts, therefore reduction of radiation exposure is indicated, if possible. Regarding exposure settings, tube voltage should be adjusted to the diagnostic scan, as pointed out previously. However, tube current may be reduced, which will increase image noise with unchanged image contrast, which is crucial for bolus tracking. Based on Brooks formula (Fig. 2), the increased collimation and slice thickness help to reduce radiation exposure and noise. Depending on the site of contrast agent delivery, various lengths of bolus tracking may be necessary. To reduce the potential increased radiation, starting bolus tracking a few seconds later or performing fewer bolus tracking images should be considered. However, in case of a small child with an accelerated circulation, these approaches risk suboptimal contrast enhancement.

Multiphase protocols, e.g., liver protocol for hepatoblastoma, result in a naturally higher radiation burden. In some clinical cases (e.g., polytrauma), images in multiple phases might be ensured with a split bolus protocol, which results in less radiation exposure [25].

Radiation-induced DNA breakages are a newly described side effect of iodinated contrast agent usage. The application of an iodinated contrast agent increases the frequency of the potential harmful DNA breakages, which is directly proportional to the injected volume [26, 27]. However, the clinical relevance of this phenomenon is unknown.

### Image reconstruction

Iterative reconstruction is a valuable tool in radiation dose reduction [28]. However, the more sophisticated model-based iterative reconstruction is time-consuming and therefore its clinical application is restricted to newer CT scanners [29]. Recently, deep learning-based image reconstruction was introduced. The principle behind this technique is to train neural networks to denoise low-dose raw data. The method is promising, but results in paediatric research are still sparse.

## Practical approach to CT protocol optimisation

The next question is how to optimise a CT scan in practice. Initially, a comparison of local radiation dose levels to diagnostic reference levels (DRLs) may be helpful. DRLs are set for representative examinations of standard-sized patients and are not expected to be exceeded. DRLs are defined either at local, national or European levels. Local DRLs are obtained from within the local health care facility or group of local health care facilities and are based on the 75th percentile values of patient dose distributions from a wide representative sample of local examinations. National DRLs are obtained from a representative sample of radiology departments in the country and provided by regulatory authorities and/or scientific societies. National DRLs are based on the third quartile of median doses for a defined clinical imaging task surveyed for standardised patient groupings. European DRLs are based on the median values of the distribution of the national DRLs for a defined clinical imaging task surveyed for standardised patient groups. Recently, European DRLs were published based on data from 47% of European countries [30]. For paediatric DRLs, patient groups are defined by weight for body examinations and by age for head examinations.

An important drawback of the DRL system is that it neglects the differences in required image quality depending on the clinical question. For example, for a head CT, better image quality is required in a case of suspected cerebral haemorrhage compared to an examination for shunt control. In the recently published European Study on Clinical Diagnostic Reference Levels for X-ray Medical Imaging (EUCLID) project, clinical question-based DRLs were introduced in adults [31]. A similar study in the paediatric setting is required.

**Fig. 5:**
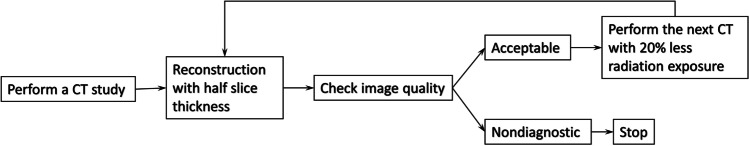
The half-slice thickness approach. *CT* computed tomography

As pointed out above, the first step in optimising local CT protocols is to define the local DRLs in a health care facility and to compare them to national or European DRLs. One should be aware that lower local DRLs in comparison to national or European DRLs do not necessarily mean the best optimisation; further adaptation of CT protocols may be required according to patient size, clinical question or available equipment. At this level, the “half thickness approach” may be helpful [32]. This can be performed without any phantoms or complicated calculations. As a first step, the operator reconstructs a standard CT scan with half-slice thickness and the paediatric radiologist assesses the image quality. If the image quality is still diagnostic, one should consider this radiation level as 100% excess dose and the next scan should be performed with 20% less radiation. This circle can be repeated until the image quality is considered nondiagnostic, which would mean that the optimal radiation level has been reached (Fig. 5).

### CT dose indices

Several dose indices are available to allow radiation exposure comparisons between different machines. In our experience, these important indicators are not well-considered by radiologists. It is therefore important that we review the main dose indices in the following paragraphs.

Volume CT dose index (CTDI_vol_) is used as the standardised measure of the radiation dose output of a CT scanner. CTDI always refers to a reference cylindrical phantom with 16-cm diameter (head) and 32-cm diameter (body). CTDI_vol_ represents the average absorbed dose to a single slice and is reported in mGy. CTDI_vol_ is a calculated value based on CTDI_w_, which is a weighted average of the measured CTDI across the field of view as follows: 1/3 CTDI in the centre and 2/3 CTDI in the edge of the cylindrical phantom. Since small children lie mostly in the centre of the table, CTDI values are prone to error in paediatric patients. However, CTDI_vol_ allows a good comparison between CT scanners and CT protocols.

To overcome the missing consideration of patient morphometry in CTDI on the resulting radiation dose, size-specific dose estimation (SSDE) was introduced. It is the best possible approximation of patient-absorbed dose in clinical practice. SSDE corrects CTDI_vol_ values by multiplying a specific conversion factor depending on the effective diameter of the patient. The effective patient diameter is measured at the largest extension of the scanned region. A known drawback of this approach is that the distinct attenuation profiles of thoracic and abdominal regions are not routinely incorporated in the calculation [33].

Dose length product (DLP) is a measure of CT tube radiation output, which considers the length of the CT scan and the radiation output along the z-axis. DLP is calculated as follows: CTDI_vol_ x length (cm). The longer the scan, the higher the radiation dose. Multiple scans also increase DLP. Since DLP calculation is based on CTDI_vol_, it inherently ignores the patient’s geometrical dimensions and therefore does not describe absorbed dose. However, k factors depending on effective body size have been published, enabling the conversion of DLP to effective dose [34].

Effective doses serve as a measure of radiation risk. Their unit is millisievert (mSv). Calculation is based on region-dependent correction factors.

### Future directions

Recently, a new photon counting detector was introduced to clinical practice. With this technology, photons are detected by a special semiconductor layer without a scintillating material, which increases the effectiveness of CT detectors and results in a promising dose reduction. This more effective detector material allows for improved spatial resolution with less electric noise and improves the detection of low energy photons that carry the most low-contrast information [35]. In our opinion, this technology might be promising in children, but to the best of our knowledge, experience of it in paediatric patients is lacking.

## Conclusion

Attracting attention to the topic of radiation protection is an ongoing effort in paediatric CT, which we paediatric radiologists need to collectively pursue. The clinical indication for CT scanning stands at the beginning of the imaging chain. Only once the radiologist has critically validated the CT request should imaging be performed, using the proper age or body-size adapted scan protocols. Contemporary equipment should be used in paediatric CT whenever possible. This manuscript summarises the relevant factors influencing dose and image quality and offers helpful hints for optimising scan parameters in clinical practice.
